# Characteristics of the complete chloroplast genome of *Swertia divaricata* Harry Sm. (Gentianaceae) and its phylogenetic inference

**DOI:** 10.1080/23802359.2023.2270211

**Published:** 2023-10-23

**Authors:** Yingying Hou, Yulong Zhang, Jing Yu, Xinjie Wang

**Affiliations:** a Department of Integrated Traditional Chinese and Western Medicine, The First Affiliated Hospital of Zhengzhou University, Zhengzhou, China; b Department of Integrated Traditional Chinese and Western Medicine, The Academy of Medical Sciences of Zhengzhou University, Zhengzhou, China

**Keywords:** *Swertia divaricata*, plastid genome, phylogenetic analysis

## Abstract

*Swertia divaricata* Harry Sm., 1965, (Gentianaceae) is a perennial herb endemic to Northwest Yunnan, China, belonging to the species-rich genus *Swertia*. It possesses unique morphological features but its systematic position remains uncertain. To determine its phylogenetic placement, the complete plastid genome of *S. divaricata* was assembled utilizing high-throughput sequencing data. The genome is circular, spanning 152,073 bp, and comprises a large single-copy (LSC) region of 82,470 bp, a small single-copy (SSC) region of 18,153 bp, and two inverted repeats (IR) regions, each 25,725 bp. A total of 130 genes were annotated, including 85 protein-coding genes, 37 tRNA genes, and eight rRNA genes. The plastome of *S. divaricata* exhibits a structure and gene composition highly similar to those of other *Swertia* plastomes. Phylogenetic analysis indicated that *S. divaricata* is closely related to *S. erythrosticta*, sister to a subclade comprising species from sections *Swertia* and *Apterae*. The plastome sequence described herein constitutes a valuable contribution to phylogenetic and evolutionary research on *Swertia*.

## Introduction

The genus *Swertia* L. (Gentianaceae) is globally distributed and comprises approximately 170 species, with the Qinghai-Tibet Plateau serving as the center of its distribution and diversity (Ho and Liu 2015). Among the 11 sections in *Swertia*, section *Swertia* represents one of the most species-rich taxa and encompasses 19 species. *Swertia* is considered one of the most taxonomically challenging genera within Gentianaceae due to paraphyletic characteristics, and the phylogenetic relationships within the genus remain contentious (Xi et al. 2014; Sun and Fu, 2019; Zhang et al. 2020a; Cao et al. 2022; Chen et al. 2023).

*Swertia divaricata* Harry Sm., 1965, a member of section *Swertia*, is highly valued in Chinese traditional medicine (Brahmachari et al. 2004; Ho and Liu 2015). *Swertia. divaricata* is endemic to Northwest Yunnan, China, and is only observed in its type locality at present (Ho and Liu 2015). The species exhibits distinct morphological traits within the genus, for example strictly divaricate inflorescence, 5-merous flowers, 1 to a few fimbriae at the base of the nectary. As it has not been previously incorporated into any molecular phylogenetic analyses, its systematic placement remains ambiguous. In this study, we characterize the full plastome of *S. divaricata* and reevaluate its phylogenetic position within the genus.

## Materials and methods

**Figure 1.**
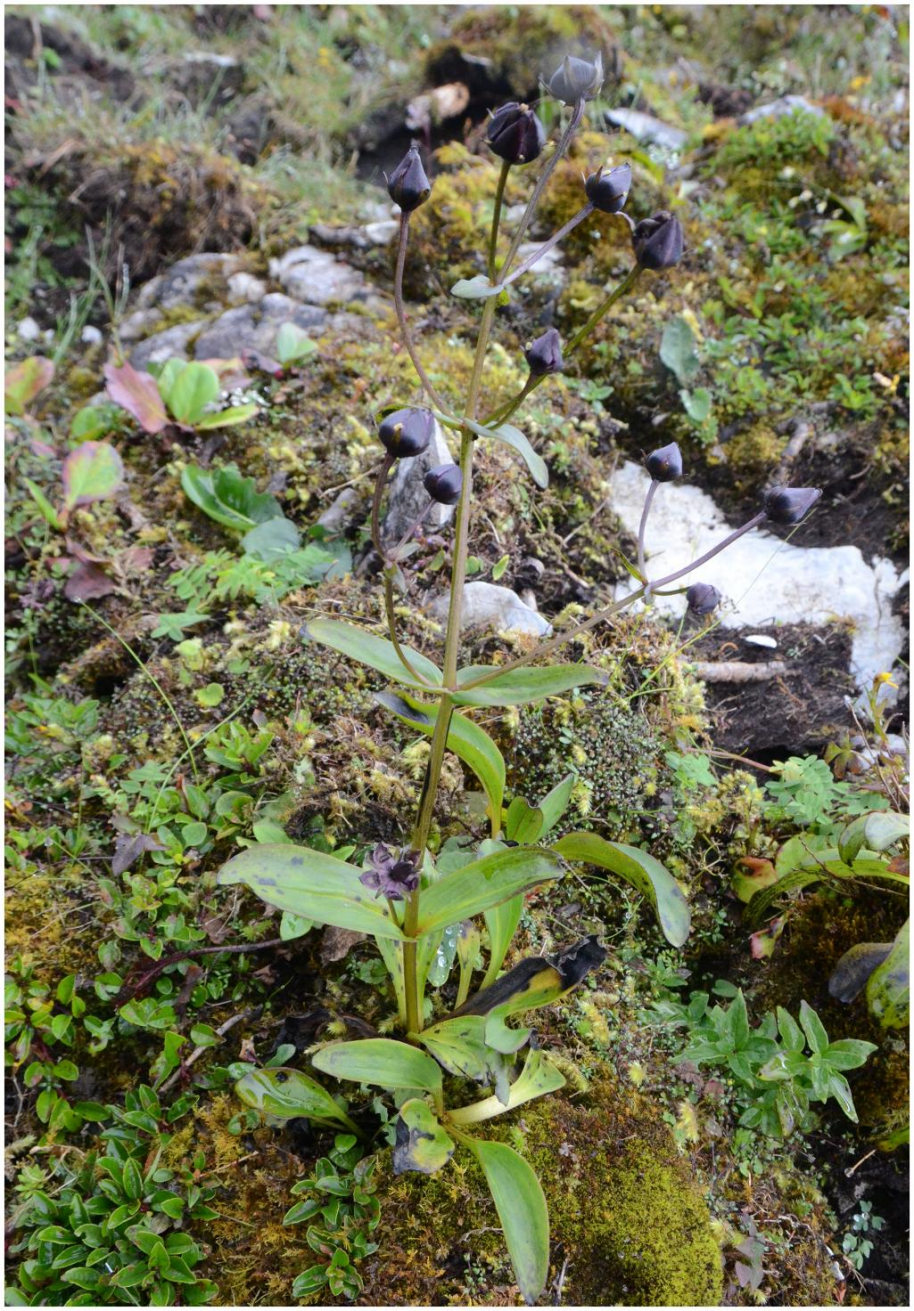
*Swertia divaricate. Swertia divaricate* is a perennial herb, has divaricate inflorescences and blackish purple corolla. Photographs by Pengcheng Fu in Gongshan, Yunnan Province, China.

We collected fresh leaves and specimens of *S. divaricata* (Figure 1) from Gongshan Town, Yunnan Province, China (28.24°N, 98.27°E). *S. divaricata* is not an endangered species, and specimen collection did not require permission. Dr. Pengcheng Fu identified and deposited the voucher specimen (Fu2018063) at the Herbarium of Luoyang Normal University (Bin Cai, 987869364@qq.com). We extracted total DNA using the Dzup plant genomic DNA extraction kit (Sangon, Shanghai, China). We constructed a paired-end library with an insert size of 150 bp and sequenced it using the Illumina HiSeq 2500 platform (Novogene, Tianjin, China). We filtered and trimmed raw reads with Trimmomatic v0.32 (Bolger et al. 2014) to eliminate adaptor sequences, low-quality reads, and sites, and checked for quality using FastQC v0.11.2. We assembled the plastome using GetOrganelle version 1.7.1 (Jin et al. 2020) and annotated it using GeSeq (Tillich et al. 2017) with default parameters. The reads coverage was plotted using a Python script (Ni et al. 2023). We drew the genome map using CPGview (http://www.1kmpg.cn/cpgview). We compared the plastome with *Swertia* species (Zhang et al. 2020a; Cao et al. 2022) and detected structural changes using mVISTA (Frazer et al. 2004). To confirm the phylogenetic position of *S. divaricata*, we extracted shared protein-coding genes from all available *Swertia* plastomes in PhyloSuite version 1.1.15 (Zhang et al. 2020b). We aligned the genes using MAFFT version 7 (Katoh and Standley 2013) and concatenated them to conduct maximum likelihood phylogenetic analyses with IQ-TREE version 1.6.12 (Nguyen et al. 2015) using 1000 replicates. We selected the best substitution model using ModelFinder 2 (Kalyaanamoorthy et al. 2017), and *Gentiana handeliana* (GenBank accession no. MN199143, Fu et al. 2021) was used as the outgroup.

## Results and discussions

**Figure 2.**
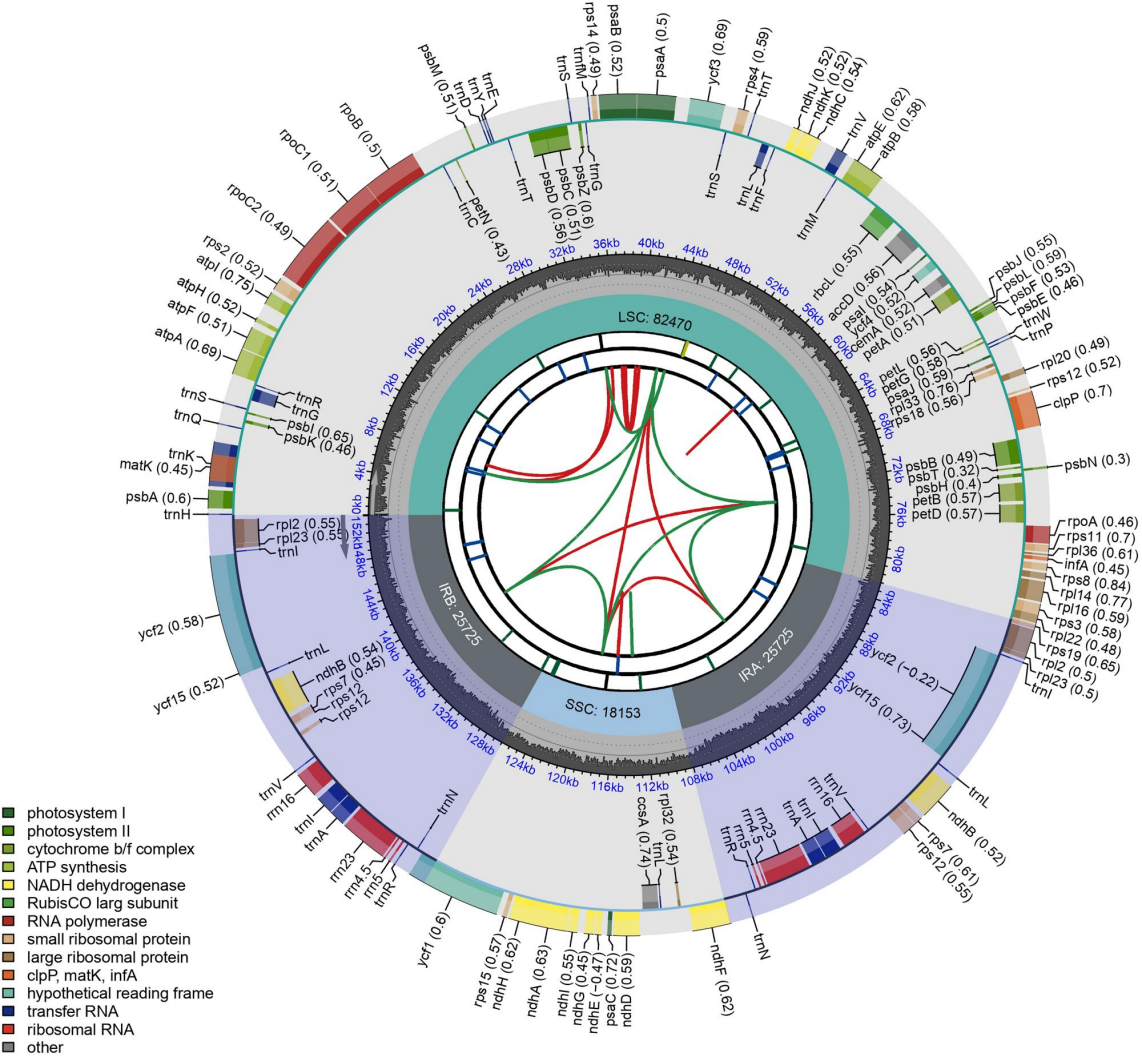
Schematic map of the chloroplast genome of *Swertia divaricata* generated using CPGview. From center outwards, first circle shows distributed repeats connected with red (forward direction) and green (reverse direction) arcs. Second circle shows tandem repeats marked with short bars. Third circle shows microsatellite sequences as short bars. Forth circle shows sizes of LSC: long single copy, SSC: short single copy regions and inverted repeat (IRa and IRb) region. Fifth circle shows GC contents along the genome. Sixth circle shows genes marked with different colors according to their functional groups.

The complete plastome of *S. divaricata* (OQ446461) is 152,073 bp long. The LSC and SSC regions are 82,471 and 18,153 bp long, respectively, and two IRs of 25,725 bp each are present. The sequencing depth ranged from 414× to 1928× and averaged at 1368× (Figure S1). We annotated a total of 130 genes, including 85 protein-coding genes, 37 tRNA genes, and eight rRNA genes (Figure 2). The 18 genes in the IR are duplicated, including 7 protein-coding genes, seven tRNA genes, and four rRNA genes (Figure 2). A total of 12 cis-splicing genes and one trans-splicing gene were identified (Figure S2). Comparative analysis showed that *S. divaricata’*s plastome structure is very similar to that in *Swertia*, and has no genes missing such as the *ndh* complex found in the subtribe Gentianinae (Sun et al. 2018; Fu et al. 2021, 2022).

**Figure 3.**
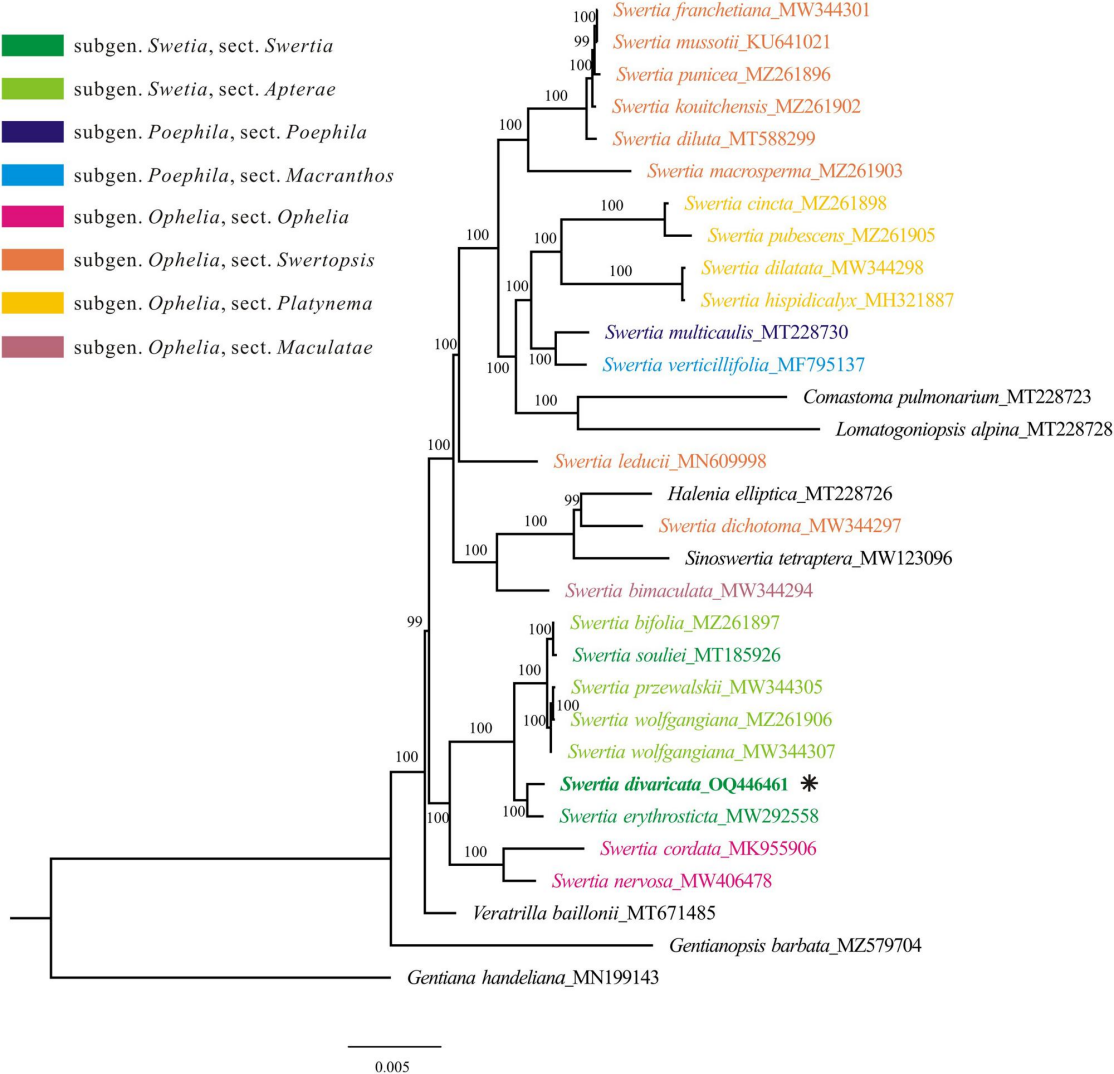
The maximum likelihood tree of *Swertia*, constructed using protein-coding genes from the plastome. Numbers above/below branches present bootstrap support. Species shown in bold is the newly sequenced in this study. The newly sequenced species was marked with an asterisk (*). The sectional classification of *Swertia* were marked with variable colors according to the treatment in Ho and Liu (2015). the following sequences were used: *S. franchetiana* MW344301 (Xu et al. 2021), *S. mussotii* KU641021 (Xiang et al. 2016), *S. punicea* MZ261896 (Cao et al. 2022), *S. kouitchensis* MZ261902 (Cao et al. 2022), *S. diluta* MT588299 (Yang et al. 2020a), *S. macrosperma* MZ261903 (Cao et al. 2022), *S. cincta* MZ261898 (Cao et al. 2022), *S. pubescens* MZ261905 (Cao et al. 2022), *S. dilatata* MW344298 (Xu et al. 2021), *S. hispidicalyx* MH321887 (unpublished), *S. multicaulis* MT228730 (Zhang et al. 2020a, 2020b), *S. verticillifolia* MF795137 (Zhang et al. 2020a, 2020b), *S. leducii* MN609998 (unpublished), *S. dichotoma* MW344297 (Xu et al. 2021), *S. bimaculata* MW344294 (Xu et al. 2021), *S. bifolia* MZ261897 (Cao et al. 2022), *S. souliei* MT185926 (Bi et al. 2020), *S. przewalskii* MW344305 (Xu et al. 2021), *S. wolfgangiana* MZ261906 (Cao et al. 2022) MW344307 (Xu et al. 2021), *S. erythrosticta* MW292558 (unpublished), *S. cordata* MK955906 (Huang et al. 2019), *S. nervosa* MW406478 (Xu et al. 2021), *Comastoma pulmonarium* MT228723 (Zhang et al. 2020a, 2020b), *Lomatogoniopsis alpina* MT228728 (Zhang et al. 2020a, 2020b), *Halenia elliptica* MT228726 (Zhang et al. 2020a, 2020b), *Sinoswertia tetraptera* MW123096 (Yang et al. 2020b), *veratrilla baillonii* MT671485 (unpublished), *Gentianopsis barbata* MZ579704 (Feng et al. 2022), *Gentiana handeliana* MN199143 (Fu et al. 2022).

Our phylogenetic analysis using a matrix of 75 protein-coding genes revealed that *Swertia* is undoubtedly paraphyletic, mixing with species from the genera *Comastoma*, *Lomatogoniopsis*, *Halenia*, and *Sinoswertia* (Figure 3). The backbone of our phylogenetic tree is consistent with that of Cao et al. (2022), and again shows the uncertainty of the generic boundary of the genus *Swertia* and its allies. One reason for the phylogenetic complexity may be the ancient origin of *Swertia* and its allies revealed by 1280 single-copy nuclear genes (Chen et al. 2023). Furthermore, our analysis indicated that *S. divaricata* is closely related to *S. erythrosticta* and is the sister clade to the clade containing species from sections *Swertia* and *Apterae*, such as *S. bifolia*, *S. przewalskii*, *S. wolfgangiana*, and *S. souliei* (Figure 3). Although *S. souliei* (section *Swertia*) is robustly clustered with section *Apterae* by our plastome data as well as single-copy nuclear genes (Chen et al. 2023), section *Apterae* including *S. souliei* is still not a monophyletic group due to chaos from species such as *S. petiolate* (Chen et al. 2023). Therefore, genomic data from more species shall be generated to explore the phylogenetic relationship in *Swertia*. This newly reported plastome sequence of *S. divaricata* provides valuable molecular data for illuminating the phylogenetic and molecular evolution of *Swertia*.

## Supplementary Material

Supplemental Material

Supplemental Material

## Data Availability

The genome sequence data that support the findings of this study are openly available in GenBank of NCBI at [https://www.ncbi.nlm.nih.gov] (https://www.ncbi.nlm.nih.gov/) under the accession no. OQ446461. The associated BioProject, Bio-Sample, and SRA numbers are PRJNA935068, SAMN33296155, and SRR23490124, respectively.
