# Supplementary figures and images for: Characteristics of the complete chloroplast genome of Swertia divaricata Harry Sm. (Gentianaceae) and its phylogenetic inference
Source: Mitochondrial DNA B Resour. 2023 Oct 23;8(10):1132–6. doi: 10.1080/23802359.2023.2270211 (PMC10621253; doi:10.1080/23802359.2023.2270211)

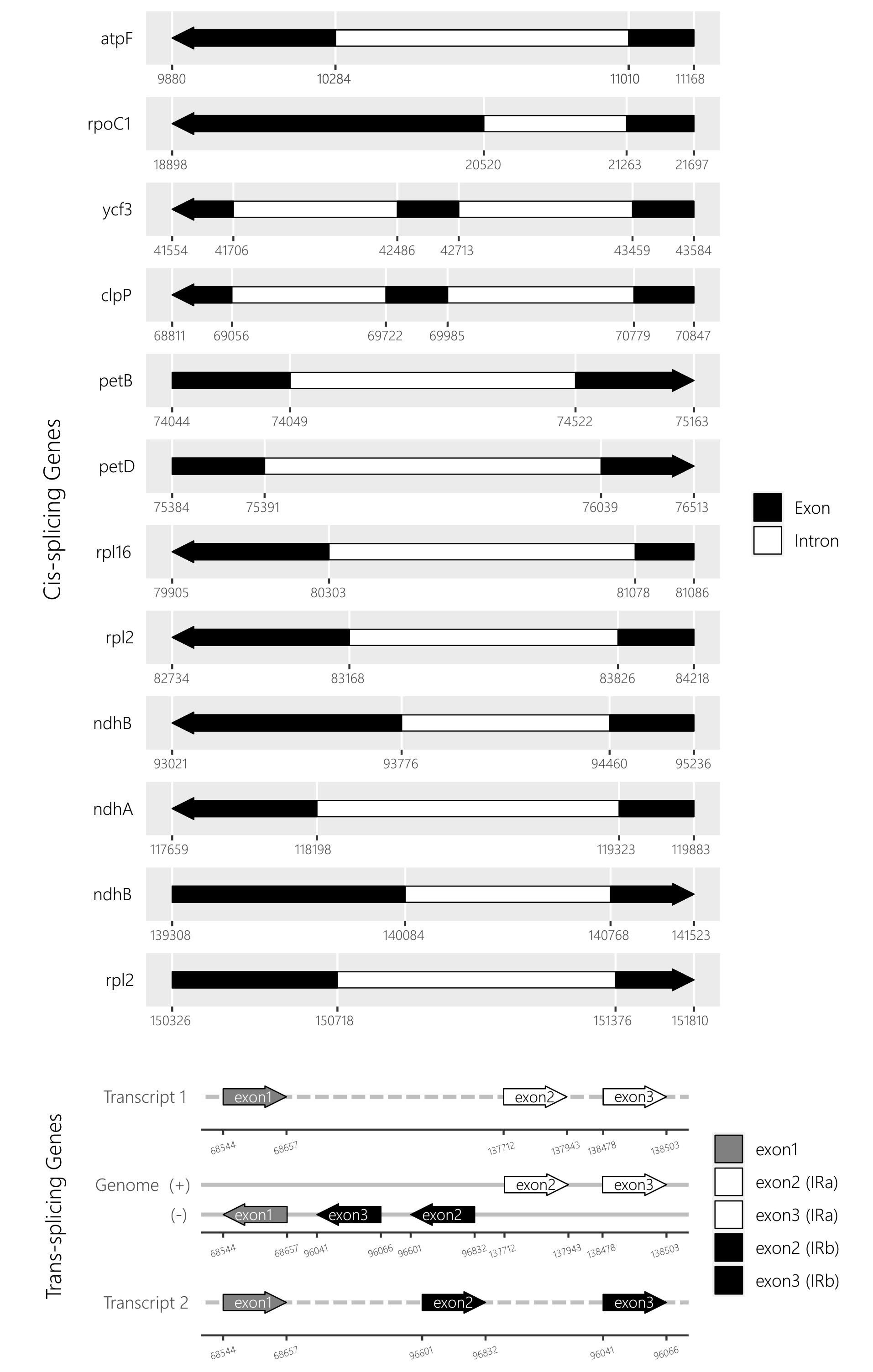

Supplement: Supplemental Material [file TMDN_A_2270211_SM1500.jpg]

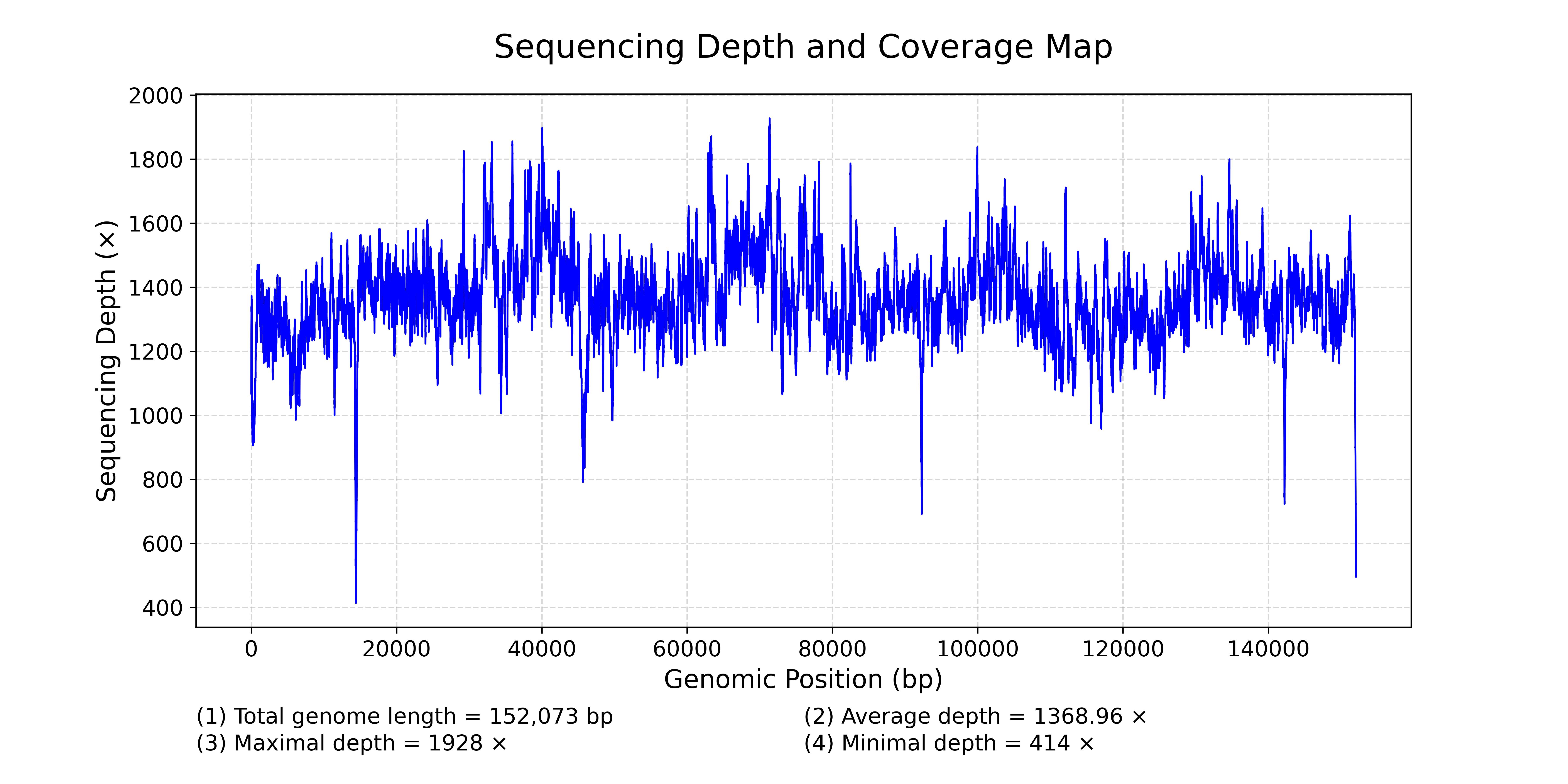

Supplement: Supplemental Material [file TMDN_A_2270211_SM1425.jpg]
